# Selection of candidate reference genes for RT-qPCR analysis in *Argulus siamensis* and their validation through screening of drugs and drug targets

**DOI:** 10.1038/s41598-019-54881-w

**Published:** 2019-12-04

**Authors:** Pramoda Kumar Sahoo, Sonali Parida, Amruta Mohapatra, Jyotirmaya Mohanty

**Affiliations:** 1 Fish Health Management Division, ICAR-Central Institute of Freshwater Aquaculture, Kausalyaganga, Bhubaneswar 751 002 India; 2 Fish Health Management Division, ICAR-Central Institute of Freshwater Aquaculture, Kausalyaganga, Bhubaneswar 751 002 India; 3 Fish Health Management Division, ICAR-Central Institute of Freshwater Aquaculture, Kausalyaganga, Bhubaneswar 751 002 India; 4 Fish Genetics & Biotechnology Division, ICAR-Central Institute of Freshwater Aquaculture, Kausalyaganga, Bhubaneswar 751 002 India

**Keywords:** Transcription, Drug screening

## Abstract

*Argulus* spp. are economically important fish ectoparasites. The development of antiparasitic drugs is thus important and real time PCR is an indispensable tool in drug development. The analytical potential of RT-PCR depends upon accurate normalisation by the use of stable reference genes. Here, we identified stable reference genes of *Argulus siamensis* for validation of efficacy of drugs and drug targets. Seven candidate genes were evaluated by evaluating their expression under different states of *Argulus* using the RefFinder tool. The four algorithms together generated a comprehensive ranking with elongation factor-1 alpha (EF-1α) being the most stable and 18S ribosomal protein (18S) the least stable gene. Taking EF-1α and 18S genes as references, the effectiveness of six anti-parasitic compounds against *Argulus* was evaluated by studying their effect on the expression pattern of few ion channel genes; this was to understand their mode of action, besides validating the reference genes. EF-1α was found to be the most stable gene in the validation. Collectively, this study is the first report to validate the optimal reference genes of *A*. *siamensis* for normalisation, and the potential of the ion channel genes for evaluating effective drug targets in parasite control.

## Introduction

*Argulus siamensis* is an economically important crustacean ectoparasite in freshwater aquaculture affecting the production of Indian major carp species (IMCs), mainly *Labeo rohita*^1^. An annual loss to the tune of 3000 million INR (625,000 US$) has been reported due to this parasite infection in Indian carp farming^1^. The parasite infects a large number of scaly fish species, with profound and underestimated effects, caused particularly by growth impairment. Not much progress has been made in the control and treatment of argulosis caused by *Argulus* spp. worldwide in both freshwater and brackish water fish. Traditionally, for the control of these parasites a variety of chemical compounds (pyrethroids and organophosphates), few anti-parasitic drugs (avermectins and moult inhibitors), and other compounds (proven at laboratory level) developed in various laboratories as anti-argulosis agents^2–7^ have been used. These methods have shown lower efficacy, cause stress in fish, and have damaging effects on the environment^8^; parasites develop gradual resistance to these drugs as well. In addition, owing to their off-target effects, the commercially available drugs have restricted use in polyculture ponds where carps are being co-cultured with crustacean prawns. Herbal extracts with anti-parasitic properties could work as environmental-friendly alternatives to anti-parasitic synthetic drugs.

Owing to its cost-effectiveness, efficacy, and eco-friendly properties, phytotherapy has gained prominence as a promising approach to combat diseases in aqua-farms^9^. Many medicinal plants extracts and essential oils have been evaluated for their anti-parasitic activity against economically important tick species with encouraging results^10–17^. Shyma *et al*.^18^ investigated the efficacy of crude extracts of garlic, *Allium sativum* cloves and *Carica papaya* seeds against *Rhipicephalus* (*Boophilus*) *microplus*. Raw garlic extract has been tested effectively against trichodinids^19^ and theronts and tomonts of *Ichthyophthirius multifiliis*^20^. Recently, effect of piperine and azadirachtin against *Argulus* spp. were also evaluated in goldfish^7^. However, the mechanisms underlying the antiparasitic activity of these herbal extracts against fish ectoparasites is poorly understood. One of the ways the anti-parasitic drugs work is through interference in the activities of ion-channel genes of the parasite neural system. Ion channel genes are important molecules involved in signal transduction in biological membranes, and are targeted by most anti-parasitic drugs and chemicals owing to some obvious advantages. Different ligand gated ion channel activity genes are the main targets for the anti-parasitic drugs like avermectins^21^. Drugs belonging to the group avermectins— doramectin and ivermectin—have been widely used to control *A*. *siamensis* infestation in carps^22^. These drugs act by binding to and blocking glutamate-gated (GluCl) and γ-amino butyric acid (GABA-Cl)-gated chloride channels in the invertebrate nervous system^23^. In *Lepeophtheirus salmonis*, avermectins cause toxicity because of their interaction with neuronal acetylcholine receptor and GABA-gated chloride channel^21^. Like avermectins, anti-parasitic herbal extracts possibly target the ion channel pathways; however, this needs to be experimentally validated.

Understanding the changes occurring in the ion channel-pathway genes under the effect of herbal extracts using mRNA expression quantification is one of the methods to validate the mode of action of the extracts. However, when using real time PCR for mRNA expression studies, use of a stable reference gene system is critical for proper normalisation of data. Further, the ion-channel genes or neuropeptides or their receptors in aquatic crustaceans have not so far been explored in lice control for developing new drug targets.

Real time polymerase chain reaction (RT-qPCR) is one of the widely used, powerful tools for detection and quantification of selected genes, for validation of transcriptome or microarray data, and for screening of drug targets. For relative quantification, the normalisation of the data with a reference gene is essential to avoid the background noise. A stable reference gene should maintain a stable expression under different experimental conditions in all tissue types tested. Thus, the selection of appropriate reference gene is a daunting task as transcript expression levels vary with physiology, pathology, and developmental stage^24^. There is no single reference gene, which is universally expressed at constant levels in all species under different experimental conditions. More than one gene has been found to have stable expression at different developmental stages, in different tissues types and under various patho-physiological conditions for a single organism. Thus, it is necessary to validate a potential reference gene for each new species before evaluating their gene expression patterns. There are different statistical algorithms such as geNorm, NormFinder and BestKeeper that have been used for the determination of candidate reference gene stability under one or several experimental conditions^25^. These programs have been used to identify stable reference genes in many plants, fish and insects.

In the present study, seven candidate genes — ribosomal protein-L32 (RP-L32), beta actin (β-actin), elongation factor-1 alpha (EF-1α), alpha tubulin (α-tubulin), ribosomal protein-S20 (RP-S20), glyceraldehyde 3-phosphate dehydrogenase (GAPDH) and 18S ribosomal RNA (18S) — were evaluated for identification of a suitable reference gene for *A*. *siamensis* obtained from different patho-physiological states. The most stable and the least stable candidate reference genes were used for expression analysis of different ion channel genes of *A*. *siamensis* such as GABA (gamma-aminobutyric acid), ICA1-4 (ion channel activator proteins 1–4), and NTR (neurotransmitters protein) in response to different herbal and anti-parasitic drug treatments. Besides establishing a reference gene in the *A*. *siamensis* system, the findings of this study add to our understanding of the possible mode of action of few anti-parasitic compounds. This study paves the way for identification of alternative drug targets in ectoparasite models.

## Material and Methods

### Parasite (*A*. *siamensis*) collection and their exposure to different treatments

A population of *A*. *siamensis* maintained on *Labeo rohita* (approx. 500 g) in 500 L fibre-reinforced plastic tank under optimal water parameters in the *Argulus* Challenge Facility of the ICAR-Central Institute of Freshwater Aquaculture, Bhubaneswar, India was used for this study. The parasites (approximately equal numbers from each growth stage) at different growth stages were collected from the infected fish and 120 numbers each were kept in five different 50 ml beakers containing aerated Milli-Q water. All collected parasites were allowed to starve for 12 h. The parasites of one beaker were washed with distilled water, and 25 numbers of each stage (matured male, female, and metanauplii) were collected separately and preserved in RNAlater in triplicates. Parasites of three other beakers were treated with deltamethrin (125 ng) (0.01 µl/L of water) (MSD Animal Health, NJ, USA, Product ID:19937557173), lipopolysaccharide from Gram-negative bacteria (LPS, Cat no. - L2880, Sigma-Aldrich, USA) (10 µg/µl of water) and Gram-positive bacteria, *Staphylococcus aureus* (10^7^ cfu, ATCC^®^, 700699, HiMedia, Mumbai) respectively, for 6 h. The parasites of the fifth beaker were used as control with no treatment. Both exposed as well as control parasites (25 numbers of parasite/group) from the four beakers were randomly collected (irrespective of size and sex) after 6 h in three 2.0 ml microfuge tubes as biological replications. These samples were washed thrice with distilled water and then stored in RNAlater for further processing. Total RNA was extracted from the parasite samples, and cDNA was synthesised using the standard protocols as described earlier^26^.

### Selection of candidate reference gene and designing of primers

Based on earlier studies^27–29^, seven reference genes—EF-1α, α-tubulin, β-actin, GAPDH, RP-L32, RP-S20 and 18S—were selected as candidate reference genes in this study. The details of these candidate genes are given in Table 1. The primers for all selected candidate genes were designed from the sequences obtained from the transcriptome data (GenBank accessions: JW945641-JW987999) of *A*. *siamensis*^30^ (Table 2). The specificity of all primers were checked and confirmed by obtaining amplicons of the desired size using semi-quantitative PCR and further sequencing of these amplicons commercially (AgriGenome, Kochi, India).

**Table 1:** The details of housekeeping genes used in this study.

Gene name	Symbol	Function
Elongation factor-1α	EF-1α	Protein synthesis
Glyceraldehyde-3-phosphate dehydrogenase	GAPDH	Enzyme involved in glycolysis
Beta actin	β-actin	Cytoskeletal protein
Ribosomal protein -S20	RP-S20	Structural component of 30 s ribosomal subunit protein
α-tubulin	AT	Cytoskeletal protein
Ribosomal protein-L32	RP-L32	Structural component of 60 s ribosomal subunit protein
18S ribosomal rRNA	18S	Structural component of ribosomal subunit protein

**Table 2:** Details of the primers used in this study.

Sl. No	Gene name	Primer Sequence (5′ → 3′)	Product size (bp)	Accession details
1	EF-1α	F-AGCCATTTATGTTGCC	129	JW947210
		R-GTTTCTTGCCATACCC		
2	GAPDH	F-TATGTTTGTTTGTGGGG	243	JW945746
		R-GAAGCAGGAATCATCTT		
3	β-actin	F- ATCTGGTGATGGTGTCTCT	140	RUN2_c81560/1/1438 (GHJM00000000)
		R- GGTGGTTGTGAAGGAGTAG		
4	RP-S20	F- TGCCAACTAAGACCTTACG	140	RUN1_c26743/2/466 (GHJM00000000)
		R- TGTCACTTGCTTAACCAGAT		
5	α-tubulin	F- GAATGAGAACTGGTACTTATCG	175	RUN2_c48193/1/1422 (GHJM00000000)
		R- GTGGAATAAGAGAATCCTTGAG		
6	RP-L32	F- TCGTCAATAACACCAAGGAA	154	RUN2_c78971/5/478 (GHJM00000000)
		R-AGTCTGGCATTAGCATTAGT		
7	18S	F-CTCGGTTCTATCTTGTTGG	200	KM597746
		R-CTGGTTGGCATCGTTTA		
8	ICA-1	F-GCACAGCCAGAACAGTATA	222	JW956493
		R-AGGATAGAGCGACACAGAA		
9	ICA-2	F-AGCGTGTCAGCATTCATTA	130	JW956551
		R-CTCAAGAAGTATTCACCTTAGC		
10	ICA-3	F-CACAGACCGTTACTTATACAG	248	JW956688
		R-GGATGCTTCATAGATAGGATTG		
11	ICA-4	F-GGTGTTGAAGTATGAGAAGAAG	234	JW956799
		R-TGTATAACTGGTGGTGGATTC		
12	NTR	F-CTACGCATACCAGTTCCTAA	264	JW958436
		R-ATCTCCTCCATCATATCCTCT		
13	GABA	F- TGGCACTTCCTTCTGTTAG	188	JW966650
		R- TTATCTCGTCGGCAATCAA		

### Quantitative real-time PCR (RT-qPCR)

RT-qPCR was performed on LightCycler 96 SW 1.1 (Roche, Germany) with FastStart Essential DNA Green Master mix (Roche, Germany) as described earlier^26^. Each sample had three biological replicates with two technical replicates. Negative control was included without adding cDNA in each assay. Prior to it, the slope of the standard curve for each gene was generated to find out PCR efficiency (E) and correlation coefficient (R^2^) using ten-fold dilution series from the pooled cDNA. The efficiency was calculated based on the following formula: E (%) = (10^−1/slope^ − 1) × 100^31–33^.

### Analysis of stability of the reference genes

Out of the seven genes, the most stable reference gene was identified using four widely used software programmes: comparative ΔCt, geNorm, NormFinder and BestKeeper^34–36^. The expression level of each gene was determined from the cycle threshold (Cq values). geNorm stability value (M) for each gene was evaluated as the average pair-wise variation for that gene; and the gene that had lower M value suggested higher gene expression stability. The stability value of NormFinder was calculated by combining inter-intra group variation determined using the excel NormFinder add-in and the genes were ranked by increasing order of stability value. BestKeeper software was used to analyse the Cq value efficiencies of each primer as input data further calculating the standard deviation (SD); a lower SD suggests high stability. Another method was comparative ΔCt, which compared the ΔCt value in two genes under different condition, and constant values in different samples indicated the stability of both genes. These four algorithms together generated a comprehensive ranking of the most sable gene among the candidate genes.

### Validation and application of stable reference gene in RT-qPCR analysis used for screening of anti-parasitic agents and their targets in *A*. *siamensis*

#### Preparation of herbal extracts

The plant materials *viz*., neem (*Azadirachta indica*) leaves (NL), papaya (*Carica papaya*) leaves (PL), matured papaya seed (PS), and garlic (*Allium sativum*) cloves (GC) were collected from the local market of Kausalyaganga, Bhubaneswar, Odisha, India. The plant materials were cleaned thoroughly and dried in shade at 25 °C, and then finely crushed using an electric grinder. The powdered samples were soaked in 70% hydromethanol at 25 °C for three days. The mixtures were shaken at 200 revolutions per minute (rpm) for 2 h followed by centrifugation at 4000 rpm for 20 min at 4 °C. The supernatant were filtered using Whatman No. 4 filter paper, the methanol was evaporated using rotary evaporator and finally the extracts were resuspended in dimethyl sulfoxide (DMSO) to yield 200 mg/ml of the extracts^37^.

#### Exposure of *A*. *siamensis* to different herbal extracts and commonly used pesticides

Plant extracts of 100 µl each (neem leaves, papaya leaves, papaya seed, and garlic cloves) were taken and mixed in 20 ml of water in a beaker, and *A*. *siamensis* parasites (35 numbers of adult collected as mentioned earlier) were treated with these extracts for 45 min. The parasites were also treated with two chemical parasiticide group of drugs, ivermectin (NEOMEC, Intas Pharamaceuticals, Ahmedabad, India) and amitraz (RIDD, Provimi Animal Nutrition India Pvt. Ltd, Bangalore, India) at a concentration of 10 µg/ml and 0.1 µg/ml, respectively, for 45 min. From each treatment group, 10 numbers of live parasites were collected in RNAlater with three biological replications. RNA extraction and cDNA synthesis from these samples were carried out as described above.

#### Effects of the above mentioned compounds on the expression of ion-channel genes of *A*. *siamensis*

Sequences of six ion channel activity genes, namely GABA (gamma-aminobutyric acid), ICA1-4 (ion channel activator proteins 1 to 4) and NTR (neurotransmitters protein) were derived from the transcriptome data of *A*. *siamensis* and primers were self-designed using Primer Premier 5 (version 5.0, Premier Biosoft International, Palo Alto, CA) (as detailed in Table 2). Expression of the above mentioned ion channel genes were analyzed by using the most stable reference gene (EF-1α) and least stable reference gene (18S) of *A*. *siamensis* based upon the results obtained in our previous experiment. Real time quantitative PCR was carried out using cDNA samples of drug/compound-treated parasites (NL, PL, GC, PS, ivermectin, amitraz) using Light Cycler 96 SW 1.1 (Roche, Germany) with FastStart Essential DNA Green Master (Roche, Germany) according to the manufacturer’s instructions^26^. All reactions were performed simultaneously for each gene with EF-1α and 18S as housekeeping genes run in triplicate. Specificity of the primers was verified by melt curve analysis of the product at a temperature of 95 °C for 10 s, 65 °C for 1 min and 95 °C for 1 min, and also by checking the size of amplicons generated in agarose gel electrophoresis. No-template controls were also included in each run. The quantification cycle (Cq) values were calculated using Light Cycler 96 SW 1.1 and the comparative Cq method by calculating the average of each Cq for triplicate samples. The Cq value of the gene for each cDNA was subtracted from its respective Cq value of reference gene to get the ∆Cq value. Since the samples for each group were taken in triplicate, an average of the ∆Cq values was obtained. Further, by subtracting the ∆Cq of the samples from the ∆Cq value of the calibrator, ∆∆Cq was calculated. Fold difference was calculated as 2^−∆∆^Cq. Mean fold difference was calculated and represented as ± standard error.

#### Statistical analysis

The expression levels of the gene in different treatment samples were compared using one-way ANOVA followed by Duncan’s multiple range tests, with values p < 0.05 as significantly different. All values of n-fold differential expression were plotted in a graph.

## Results

### Primer specificity, PCR efficiency, and expression profile of the candidate reference genes

A **s**ingle PCR product of expected size from each primer pair of candidate reference genes was amplified in agarose gel electrophoresis (Supplementary Fig. S1). Amplification specificity was also confirmed by melt curve analysis with single peak for each candidate reference gene (Supplementary Fig. S2) followed by sequence analysis. Standard curves were generated using serial dilution series, along with high linear correlations (R^2^ > 0.96) for all genes. The PCR efficiencies for seven different genes varied from 99% to 119% (Table 3).

**Table 3:** Amplicon characteristics of candidate reference genes.

Gene	Linear regression equation	Correlation (R^2^)	Amplification efficiency (%)	Slope
RP-L32	−2.864x + 36.67	0.97	118	−2.864
β-actin	−3.333x + 33.37	0.99	99.52	−3.333
EF-1α	−3.198x + 37.89	0.97	105	−3.198
α-tubulin	−2.929x + 41	0.96	119	−2.929
RP-S20	−3.337x + 38.89	0.98	99	−3.337
GAPDH	−3.331x + 31.42	0.99	99	−3.331
18S	−2.876x + 36.13	0.97	102	−2.876

The expression level of the candidate reference genes was determined by RT-qPCR across various physiological states (adult stages of male and female, metanauplii), and pathological states (i.e., deltamethrin-treated, LPS and gram positive bacteria-induced) of the parasite. The candidate reference genes displayed wide range of accumulation level across all the tested samples, with threshold cycle (Cq) values spanning 8.04 to 33.78 (Table 4). The lowest expressed reference gene was 18S (mean Cq = 8.04, SE = 0.01) in Gram positive bacteria induced adult parasite, while α-tubulin showed the highest expression level (mean = 33.78, SE = 0.78) in adult female parasite.

**Table 4:** Cq values of reference genes (mean ± SE) at different patho-physiological states of the parasite *A*. *siamensis*.

Gene	Physiological states of the parasite			Pathological states of the parasite		
	Adult male	Adult female	Metanauplii	Pesticide-deltamethrin treated adult parasite	*Staphylococcus aureus-* treated adult parasite	LPS- treated adult parasite
EF-1α	23.45 ± 0.26	25.60 ± 0.85	22.67 ± 0.27	23.64 ± 0.25	21.22 ± 0.04	21.31 ± 0.02
GAPDH	23.20 ± 1.62	19.41 ± 0.13	18.84 ± 0.4	20.06 ± 0.17	19.80 ± 0.01	20.01 ± 0.02
β-actin	19.45 ± 1.62	17.85 ± 0.22	17.34 ± 0.52	18.35 ± 0.15	17.04 ± 0.05	17.77 ± 0.60
RP-S20	27.54 ± 0.65	22.13 ± 0.22	21.38 ± 0.99	27.87 ± 0.15	22.03 ± 0.29	21.06 ± 0.02
α-tubulin	30.83 ± 0.54	33.78 ± 0.78	29.46 ± 0.20	29.97 ± 0.31	30.08 ± 0.04	30.45 ± 0.04
RP-L32	25.97 ± 0.43	26.72 ± 1.70	21.42 ± 0.19	21.91 ± 0.16	30.08 ± 0.07	30.45 ± 0.10
18S	22.54 ± 3.52	10.85 ± 0.300	9.88 ± 0.50	9.29 ± 0.15	8.04 ± 0.01	9.03 ± 0.02

### Expression stability of candidate reference genes

**Figure 1:**
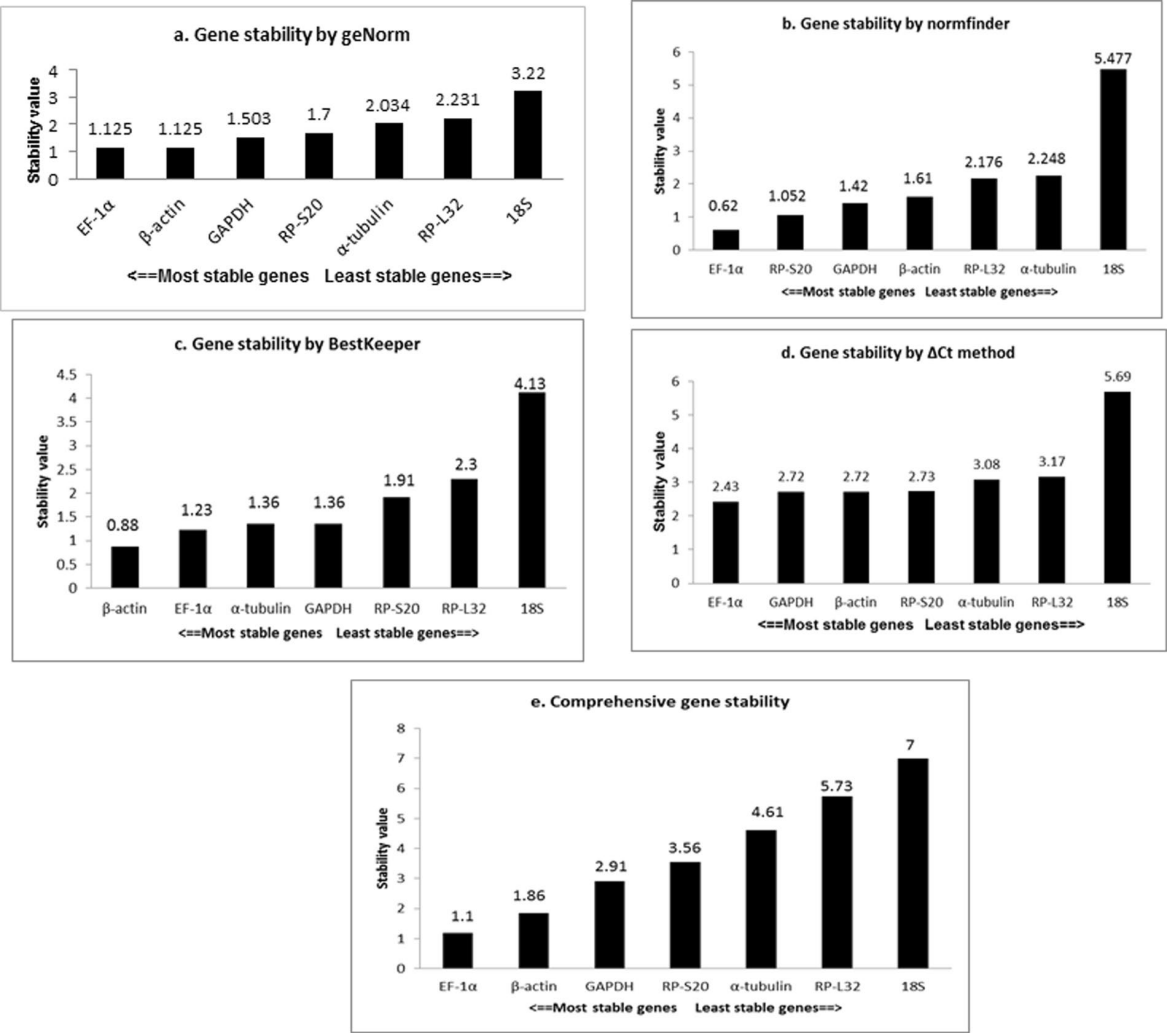
The stability value of seven candidate reference genes calculated by (**a**) geNorm (**b**) NormFinder, (**c**) BestKeeper, (**d**) Comparative ΔCt and (**e**) comprehensive ranking in seven different patho-physiological conditions.

The expression stability across different patho-physiological states of the parasite was determined using four methods (geNorm, NormFinder, BestKeeper and ΔCt). Among the seven candidate reference genes, geNorm analysis indicated that EF-1α and β-actin were the most stable genes, and RP-L32 and 18S as the most unstable genes (Fig. 1a). As per NormFinder analysis the rank of seven candidate genes from most stable to least stable genes were as EF-1α, RP-S20, GAPDH, β-actin, RP-L32, α-tubulin and 18S (Fig. 1b). In BestKeeper the two most stable genes found were β-actin and EF-1α, and the other genes in the rank from most stable to unstable were observed as α-tubulin, GAPDH, RP-S20, RP-L32 and 18S (Fig. 1c). According to comparative ΔCt method algorithm, EF-1α and GAPDH were found to be the most stable genes while RP-L32 and 18S as the most unstable genes (Fig. 1d).

### Comprehensive ranking

A comprehensive ranking was generated by combining these four algorithms by a tool available online (RefFinder tool) (Fig. 1e). Based on this comprehensive ranking, EF-1α and β-actin were found to be the most stable candidate reference genes for *A*. *siamensis* whereas 18S and RP-L32 were the least stable genes. Further, EF-1α was found to be the least variable candidate gene with a coefficient of variation (CV) of 0.20 to 8.10% followed by β-actin (CV ranging from 0.82 to 8.30%), and 18S showed the highest variability, with CV ranging from 0.60 to 38.20% in different states of parasite. The pair-wise variation (V) calculated by geNorm method indicated either EF-1α or β-actin could be used for reliable normalisation.

### Validation of reference gene- adding to the knowledge of mechanism of action of herbal and chemical anti-parasitic agents on parasite ion-channel genes

**Figure 2:**
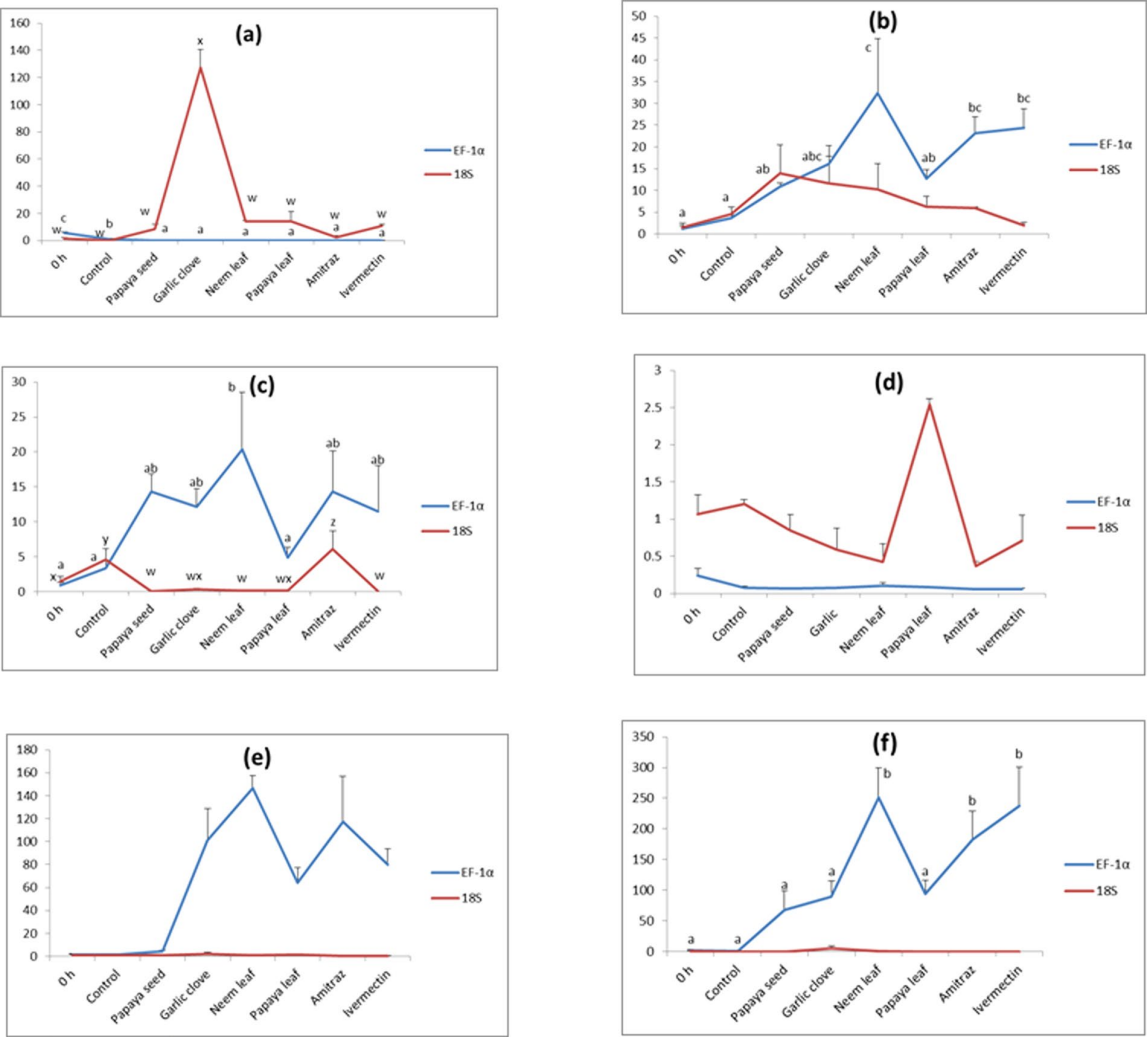
Relative gene expression study of six ion channel genes (**a**) ICA-1, (**b**) ICA-2, (**c**) ICA-3, (**d**) ICA-4, (**e**) NTR, (**f**) GABA in different parasite samples treated with four herbal extracts (PS, GC, NL and PL), ivermectin and amitraz using most stable (EF-1α) and least stable reference genes (18S) for normalisation. Significant difference (P < 0.05) of gene transcript levels among the samples using either EF-1α or 18S as reference gene were indicated by a, b, c, d, or w, x, y, z, respectively on top of the standard error bar. Y-axis represents fold-expression.

The expression of six ion channel activity genes has been studied in different herbal, ivermectin and amitraz treated parasite samples. The relative qPCR gene expression study was carried out using the most stable EF-1α and the least stable 18S as reference genes in normalisation for their further validation (Figs. 2a–f). The expression of ICA-1 gene was found to be significantly reduced in all the treated samples as compared to the 0 h and control sample collected after 45 min using EF-1α as normalisation factor where as significant increase in expression pattern was observed in garlic clove treated samples (120-fold) using 18S as internal control (Fig. 2a). The expression of ICA-2 gene was significantly upregulated (EF-1α as control) under different parasiticide treatments as compared to 0 h and 45 min control parasite samples. However, the expression was up-regulated to the significant effect of 25-fold as compared to 0 h control in NL-treated sample after 45 min exposure of the compound to the parasite (Fig. 2b). No significant up-regulation of ICA-2 gene was observed in any of the treated samples when 18S gene was taken for normalisation. Similar interesting result was also noticed in case of ICA-3 expression that was 17-fold up-regulated in NL-treated parasite as compared to its 0 h control, although the expression was high in all parasiticide-treated *Argulus* (Fig. 2c). Other parasiticide chemicals or compounds viz., GC, PS, ivermectin and amitraz could able to upregulate the expression of ICA-3 up to 10-fold as compared to 0 h control sample. Significant down regulation was noticed in expression profile of ICA-3 gene, in all parasiticide-treated *Argulus*, except amitraz treated sample as compared to control when 18S was used as internal control. The expression of ICA-4 gene was significantly reduced in all treated parasites along with 45 min control sample as compared to 0-h control parasites (EF-1α as internal control) whereas significant increase in expression was noticed in papaya leaf treated sample (3-fold) using 18S as normalisation gene (Fig. 2d). On the other hand, the expression of NT gene was highly upregulated in all treated parasiticide samples except PS-treatment and the expression as high as 150-fold was noticed in NL treatment. The expression of NT gene was 100-fold high in GC and amitraz-treated samples, and 75-fold high in PL and ivermectin-treated parasites as compared to control (Fig. 2e). Similarly, the expression of GABA gene was significantly upregulated in all parasiticide-treated samples. An up-regulation to the level of 250-fold was noticed post-NL treatment, 230-fold with ivermectin and 200-fold after amitraz treatment in the expression of GABA gene, which was quite interesting (Fig. 2f). No changes in expression pattern were observed in NT and GABA genes when unstable 18S gene was used as reference control (Table 5).

**Table 5:** Fold expression of six ion channel genes i.e., ICA-1, ICA-2, ICA-3, ICA-4, NTR and GABA in parasite sample treated with four herbal extracts (PS, GC, NL and PL), ivermectin and amitraz using most stable (EF-1α) and least stable reference genes (18S) for normalisation.

Treatment	Gene name											
	ICA-1		ICA-2		ICA-3		ICA-4		NT		GABA	
	EF-1α	18S	EF-1α	18S	EF-1α	18S	EF-1α	18S	EF-1α	18S	EF-1α	18S
Control (45 min)	4.79↓	1.25↓	2.45↑	3.05↑	2.37↑	3.13↑	0.16↓	0.13↓	0.006↑	0.47↓	1.93↓	1.01↓
Papaya seed	5.18↓	7.28↑	9.68↑	12.43↑	13.37↑	1.44↓	0.18↓	0.22↓	2.90↑	0.81↓	65.90↑	1.03↓
Garlic clove	5.23↓	126.07↑	14.91↑	10.15↑	11.16↑	1.10↓	0.17↓	0.48↓	99.42↑	1.04↑	87.53↑	3.66↑
Neem leaf	5.36↓	12.79↑	31.16↑	8.69↑	19.40↑	1.38↓	0.14↓	0.64↓	145.11↑	0.27↓	248.94↑	0.87↓
Papaya leaf	5.35↓	13.22↑	11.56↑	4.81↑	3.91↑	1.29↓	0.15↓	1.47	62.57↑	0.15↑	91.35↑	1.02↓
Amitraz	5.31↓	1.15↑	22.02↑	4.38↑	13.35↑	4.64↓	0.18↓	0.69↓	115.70↑	0.53↓	180.31↑	1.01↓
Ivermectin	5.38↓	9.70↑	23.19↑	0.49↑	10.55↑	1.45↓	0.18↓	0.35↓	78.24↑	0.85↓	235.80↑	1.03↓

## Discussion

Quantification of gene expression is an essential aspect of its functional characterisation in any species. Many methods such as Northern blotting, ribonuclease protection assay, semi-qPCR, real time PCR, molecular *in situ* hybridisation, and cDNA microarray are used to study quantitative gene expression. Among these methods, real-time PCR is considered as the most accurate, reliable, cost-effective and rapid way to analyse transcript levels of a gene^38–40^. Obtaining accurate results using real-time PCR depends on the proper selection and normalisation of reference gene^34,41^. A reference gene is stably expressed at a constant level under different patho-physiological conditions. There are many reports available indicating that there is no single gene, which is expressed stably under different experimental conditions; for example, the expression pattern of GAPDH (most commonly used reference gene) varies in triatomines intestinal tissue after blood intake^28^. Similarly, the expression of GAPDH has been shown to be relatively higher in tumour cells as compared to healthy cells. Other than GAPDH, expression of two more commonly used housekeeping genes, β-actin and ribosomal protein L30, has been shown to reduce in mononuclear cells of peripheral blood after cadmium exposure^42^. Therefore, it is suggested that only under non-cytotoxic experimental conditions housekeeping genes can be used as reference genes^42^.

Recently, many algorithms are being used for evaluation of stable gene expression. The most commonly used softwares are geNorm, BestKeeper, NormFinder and comparison of ΔCt value obtained from PCR. Comparative ΔCt compares the relative expression between two genes in different conditions, and if the ΔCt value remains constant, then it indicates that the genes are stably expressed. If the ΔCt value varies under different conditions then one of the genes is unstable^36^. Likewise, BestKeeper software analyses the stable candidate gene by using pair-wise correlation and comparing standard deviation of all individual Cq values^35^. The geNorm software is employed as a mean for determining the expression stability value (M) for each candidate gene based on the average pair wise variation between all genes analysed. The gene with the lowest M value is considered to have the most stable expression, while that with the highest M value has the least stable expression^33^. The NormFinder algorithm is used to identify the optimal normalisation gene among a set of candidate genes using both inter and intra group variations^34^. In the present study, the stable internal control was identified and validated for one of the most important fish ectoparasites, *Argulus siamensis*, which causes economic losses to the tune of 29,524.40 INR/ha/year in freshwater fish culture^1^.

Sequence information on this species is very minimal and earlier reports pertaining to expression analysis in *A*. *siamensis* have not considered a comparative analysis of reference genes. Therefore, for identification of a stable reference gene, seven most commonly used reference genes (EF-1α, α-tubulin, β-actin, GAPDH, RP-L32, RP-S20 and 18S ribosomal protein) sequences were obtained from the transcriptome of *A*. *siamensis*^30^ and used in this study. Four different algorithms generated different stable genes for *A*. *siamensis*. Stability of these seven genes were analysed using the comparative ΔCt method where EF-1α gene (stability value: 2.43) was found to be the most stable one followed by GAPDH and β-actin (stability value: 2.72). RP-S20 also showed a little more variation with stability value of 2.73. Similarly, when using NormFinder, EF-1α gene was found to be the most stable one with stability value of 0.62 followed by RP-S20. In case of geNorm analysis, both EF-1α and β-actin revealed similar stability value followed by GAPDH whereas, β-actin found to be the most stable one followed by the EF-1α in BestKeeper. As different genes were predicted to be stable by different algorithms without consensus, a comprehensive ranking for the genes was done by taking into account the results predicted by all the four algorithms and EF-1α gene was found to be the most stable gene followed by β-actin.

EF-1α is an important factor for protein synthesis, which transports aminoacyl-tRNA to the 80 S ribosome site; it also helps in signal transduction, cell growth and proliferation, mitotic apparatus formation, and arrangement of the cytoskeleton^43–45^. EF-1α is one commonly used internal control in many experiments conducted in humans, rice, mice, and insects^46^. EF-1α is the best internal control in case of *Hippodamia convergens*, an insect and its transcripts levels were constant when tested in different developmental stages, tissue types, sex and under three abiotic stress conditions^47^. In our study also EF-1α was found to be the most stably expressed gene in different developmental stages, sex and under biotic or abiotic stress exposure.

Another most commonly used internal control is β-actin, which is a structural protein involved in cell motility, formation of cell structure and its integrity. This gene has also been used as the reference gene in numerous species as human, rice, cotton and many insects such as *Apis mellifera*, *Schistocerca gregaria*, *Drosophila melanogaster*, *Plutella xylostella*, *Chilo suppressalis*, *Chortoicetes terminifera*, *Liriomyza trifolii*, and *Diuraphis noxia*^48–54^. Herein, among the seven reference genes, β-actin ranked second in comprehensive ranking. Therefore, either one or both of the above-mentioned genes emerged as potential candidates to be used as reference genes for any further expression analysis study in *A*. *siamensis*, once validated. Further, these two genes could be used in any toxicity studies as evident from their stability after exposure to biotic (bacterial) or abiotic (drug) stressors.

Additionally, to determine the influence of different reference genes in normalisation (as a process of validation), we used the most stable (EF-1α) and the least stable (18S) genes while studying the expression of six ion-channel genes of *A siamensis* following exposure to various anti-parasitic compounds. The results obtained demonstrated the significant difference in expression pattern of target transcripts when one stable and one unstable genes were used to normalise the data; thus, it was validated that use of an inappropriate reference gene could lead to misinterpretation of obtained experimental data. Hence, the results obtained only from the normalized data using the most stable gene i.e. EF-1α was further discussed here while analysing the expression pattern of ion-channel genes with respect to anti-parasitic effects.

One of the objectives of this study is to validate the evaluated reference genes found here. Here we used the reference gene in both validating and understanding the expression of ion channel genes to study the mode of action of antiparasitic compounds on *Argulus* parasites. Most of the anti-parasitic drugs target ion-channel genes of the parasite neural system, however, there is paucity of literature on how the herbal compounds influence parasite signal transduction molecules in biological membranes.

*A*. *siamensis* causes heavy economic loss in carp farming due to the absence of appropriate control measures. Several chemicals and anti-parasitic drugs are in use, but they create a very stressful condition for the fish as well as the environment with low efficacy^8^; development of drug resistance in the parasites is another shortcoming. Herbal extracts are good alternatives to chemical drugs because they are biodegradable, eco-friendly, and cost-effective. Many commonly used herbal extracts such as those of neem, garlic, papaya, green tea, and turmeric have already been proved to have anti-parasitic activities^10–18^. Crude extracts of *Allium sativum*, *Carica papaya* seeds and garlic have been tested successfully against *Rhipicephalus* (*Boophilus*) *microplus* and different developmental stages of *I*. *multifiliis*, respectively^19,20^. Potential anti-parasitic effect of *Ocimum gratissimum* was tested against *Argulus* spp. and bath treatment with crude plant extracts resulted in significant reduction of parasite count in Nile tilapia males^6^. Another effective anti-parasitic compound piperine, a compound isolated from black pepper, was tested against *Argulus* spp. in gold fish^9^. Similarly, azadirachtin, a compound isolated from *Azadirachta indica*, has anti-parasitic role against *Argulus* spp. in *Carassius auratus* and also is reported to shift serum biochemical parameters towards homeostasis^9,55^.

Although anti-parasitic properties of many plant extracts and isolated natural products have been tested *in vivo* and *in vitro*, their target sites, mode of action and downstream effects in host remain unexplored. Most of the anti-parasitic drugs and chemicals act on signal transduction through the ion channel genes, damage DNA or RNA of the parasite, degrade the important proteins or enzymes, or disturb permeability of the parasite membrane. The commonly used anti-parasitic drug for external and internal parasites of human, sheep, cow, horse, and aquatic organisms is avermectin^56^. SLICE, the anti-sea louse drug used for control of sea louse in Atlantic salmon, contains the avermectin compound emamectin benzoate^57^. Other two drugs of this group, doramectin and ivermectin, have been commonly used against *A*. *siamensis* infection in carps^22^. These drugs mainly block glutamate-gated (GluCl) and γ-amino butyric acid (GABA-Cl)-gated chloride channels of the parasite by mediating the contraction or relaxation phase of parasite muscular movement causing flaccid paralysis of the worm^23^. Here for the first time, study has been undertaken to elucidate the effect of herbal anti-parasitic agents on ion-channels genes in an aquatic ectoparasite of crustacean group.

Six different ion channels genes and neurotransmitter proteins (GABA, ICA1-4 and NTR) were selected, among which the functional existence of five genes, excluding that of GABA, was reported in a fish parasite system for the first time. Although ion channels have proven to be potential as targets for anti-parasitic agents, other components of the parasite neuronal signalling system might also serve as a fertile ground for new therapeutics^58^. Neurotransmitter transporters, important for regulating transmission, are validated drug targets in humans. Further, invertebrates signal via neuropeptides and peptide receptors for neuromuscular function, and these molecules could serve as efficient drug targets, not evident in vertebrates^58^. Up-regulation of the GABA gene was noticed at a similar level in neem leaf- and ivermectin-treated parasites as compared to the control. It has already been reported that, salmon lice with higher transcripts level of GABA are susceptible to anti-parasitic drugs as compared to resistant one^21^. Ivermectin is well-known to activate glutamate-gated chloride channels by binding to ion-channels and the process is slow but irreversible^59^, leading to channel opening and hyperpolarisation of the cell- rendering it not further excitable^60^. In this study, the ICA-2 and ICA-3 genes were found to be upregulated after neem leaf extract treatment followed by garlic clove extract and papaya seed treatments, respectively. It is clear that the herbal extracts studied here also act on the parasite either by activation of GABA or ion channels, as is the case in ivermectin or macrocyclic lactone, thus leading to influx of chloride ions into cells and paralytic death of parasites by hyperpolarisation of nerve endings. On the other hand, ICA-1 expression was significantly down regulated in all treated groups as compared to control or 0 h sample, and thus ICA-1 can also be regarded as a screening target for drug development against this parasite as an antagonist. The down-regulation of ICA-1 might result in excitation and subsequent paralysis by disruption of neuronal signal; however, this needs to be further explored for better understanding of the alternate mode of action of these compounds. All genes except ICA-4 showed significant up or down regulation in neem leaf extract treated groups and expression fold was similar or higher as compared to the two groups treated with proven chemical anti-parasitic drugs (amitraz and ivermectin). Hence, it is understood that these anti-parasitic agents kill the parasite by modulating their action on several ion channel genes including GABA at a time. However, all these genes need to be fully characterised in this crustacean parasite to understand their functional existence and to be used further in developing drug targets. Same is the case with all these anti-parasitic agents in upregulating the expression of neuropeptide studied here. Many of the neuropeptides are not found in mammals, and hence, the peptidergic signalling by these molecules could be considered a promising target for newer drug discovery^61^. At this juncture, no neuropeptide system-targeting lead compounds have been detected in crustacean parasites. It needs further study to understand any cross-talk that exists among these proteins and ion-channels, and other downstream signalling pathways. Thus in turn the herbal extracts may serve as broad spectrum anti-parasitic agents through influencing neuronal transmission or ion channel molecules. As the ion channels are highly heterogenous and complex, this study proved the functional existence of these channels in *Argulus* by showing response to different drugs and herbal extracts. The interesting fact is that when the expression of all six genes were analysed using the least stable gene 18S as internal control, their expression pattern were either over or under estimated, as expected.

This study is the first report of systematic identification and validation of candidate reference genes for normalisation during real-time PCR under a wide range of experimental patho-physiological conditions in *A*. *siamensis*. The stability of the reference genes was evaluated using four common algorithms - comparative ΔCt, geNorm, NormFinder and BestKeeper, and comprehensive ranking was done to find out the most stable reference gene for *A*. *siamensis*. EF-1α was found to be the most stable gene followed by β-actin in *A*. *siamensis*. Further, the effect of different herbal anti-parasitic extracts on the ion-channel genes of the parasite was elucidated for the first time. Based on the expression analysis of the ion-channel genes, neem leaf extract may be considered an alternative to synthetic drugs. Upregulation of most of the ion-channel genes except ICA-1 in parasites treated with herbal extracts is indicative of their effect on signal transduction in *A*. *siamensis*. The findings of this study may pave the way for development of effective, alternative drugs for *A*. *siamensis*.

## Supplementary information

Supplementary information
