# Supplementary material for: Selection of candidate reference genes for RT-qPCR analysis in Argulus siamensis and their validation through screening of drugs and drug targets
Source: Sci Rep. 2019 Dec 4;9:18365. doi: 10.1038/s41598-019-54881-w (PMC6892791; doi:10.1038/s41598-019-54881-w)
Supplement: Supplementary file 1 — Supplementary information [file 41598_2019_54881_MOESM1_ESM.docx]

Supplementary file

Selection of candidate reference genes for RT-qPCR analysis in *Argulus siamensis* and their validation through screening of drugs and drug targets

Pramoda Kumar Sahoo^1^*, Sonali Parida^2^, Amruta Mohapatra^3^ & Jyotirmaya Mohanty^4^

*ICAR-Central Institute of Freshwater Aquaculture*

*Kausalyaganga, Bhubaneswar751 002, India*

*Corresponding author (email: [pksahoo1@hotmail.com](mailto:pksahoo1@hotmail.com))


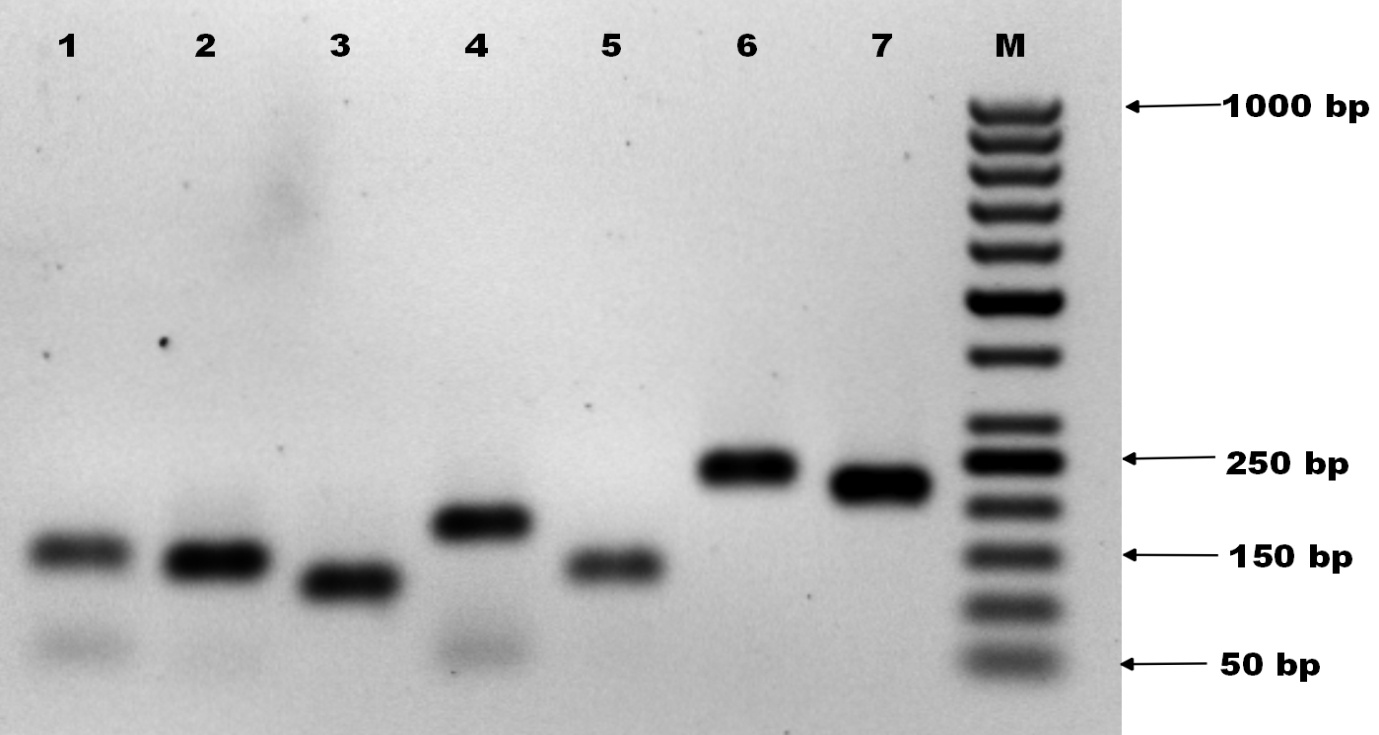


**Fig. S1.** Specific amplicons of expected size for each reference gene after RT-qPCR followed by agarose gel electrophoresis. M represents the DNA marker. Lanes1-7 represent RP-L32, β-actin, EF-1α, α-tubulin, RP-S20, GAPDH and 18S.

**Fig. S2.** Melt curve of seven candidate reference genes
